# What’s in the Powder? Evaluating Fentanyl Test Strip Sensitivity to Common Household Items in Chemical Emergency Response Scenarios

**DOI:** 10.1021/acs.chas.5c00103

**Published:** 2025-09-18

**Authors:** Kate Y. Mongold, Meshel A. Lange, Mason Shields, Heather M. Barkholtz

**Affiliations:** † Forensic Toxicology, Environmental Health Division, Wisconsin State Laboratory of Hygiene, 2601 Agriculture Dr., Madison, Wisconsin 53718, United States; ‡ Molecular and Environmental Toxicology, 37360 University of Wisconsin-Madison, 1300 University Avenue, Madison, Wisconsin 53706, United States; § Chemical Emergency Response, Environmental Health Division, Wisconsin State Laboratory of Hygiene, 2601 Agriculture Dr., Madison, Wisconsin 53718, United States; ∥ Pharmaceutical Sciences, School of Pharmacy, 37360 University of Wisconsin-Madison, 777 Highland Avenue, Madison, Wisconsin 53705, United States

**Keywords:** fentanyl, immunoassay test strips, unknown powder testing, diluents, limit of detection

## Abstract

The increasing prevalence of opioid misuse, particularly the adulteration of illicit substances with fentanyl, has heightened the need for effective field detection methods for unknown powders. First responders, including chemical emergency response and Hazardous Materials (HazMat) teams, face significant challenges in assessing chemical threats in real-time without the resources of a controlled laboratory environment. Immunoassay test strips are commonly used for drug detection and are considered potential tools for identifying fentanyl in emergency scenarios. However, the impact of common diluents (substances such as sugar, flour, and other commonplace additives) on the accuracy of these test strips is unexplored. This study evaluates the limit of detection (LOD) of a popular commercially available fentanyl test strip (FTS) in the presence of various diluents commonly found in emergency situations. The experimental LOD was determined to be 0.05 μg/mL, significantly lower than the reported LOD of 0.20 μg/mL. While no false positives were observed with diluents alone, the presence of diluents in fentanyl solutions altered FTS results, requiring higher concentrations of fentanyl to achieve a positive reading. Results were often difficult to interpret, particularly near the LOD, which could pose challenges for first responders in real-world scenarios. This work is the first to assess the application of FTS to chemical emergency response situations and threat assessment of unknown powders. Commercially available FTS are cost-effective, user-friendly, provide rapid results, and detect low concentrations of fentanyl even in the presence of other substances.

## Graphical abstract

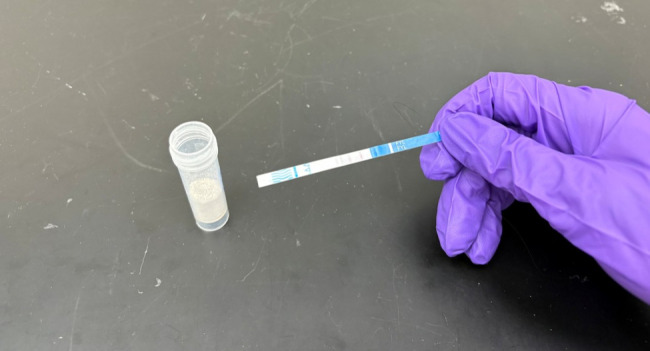

## 1 Introduction

The federal government receives up to 12 reports of hazardous substance releases a day, where in most cases, those who report are also the individuals that discovered the initial hazard.^1^ Prioritizing the safety of those who are first exposed to chemical threats is paramount – whether they are first responders, Hazardous Material Response Teams (HazMat), or members of the public. In cases of unknown spills or powders, being able to detect threats at the time of discovery is essential. These unknown powders can contain anything from illicit drugs to more benign diluents that resemble dangerous substances. These chemicals can include adulterants, diluents, and contaminants. According to the Canadian Centre on Substance Use and Addiction (CCSA), adulterants are defined as pharmacologically active ingredients added to enhance or mimic the effects of the expected substance.^2^ This can include unregulated drugs such as fentanyl, xylazine, or benzodiazepines. Diluents are inert substances added to increase the perceived volume or weight of a drug product.^2^ This can include materials with similar visual characteristics to the unknown powder in question, such as sugar, talcum powder, or flour. Contaminants are typically unintentionally introduced substances, such as unreacted starting materials or byproducts of the manufacturing process.^2^

Synthetic opioids are a common and frequently highlighted in media coverage for their role addiction and overdose deaths. Fentanyl, a central drug in the opioid epidemic, is well-known as a street narcotic, but it is also used effectively as a clinical analgesic or anesthetic in the U.S. and as an incapacitating agent in other countries.^3^ In the case of narcotics, exposure to only 2–3 mg of fentanyl can lead to overdose symptoms and possibly death.^4^ This drug is also challenging to detect because it comes in many forms (liquids, powder, pills, etc.), making it more easily disguised.^4^ It is important to note that small amounts of fentanyl are unlikely to cause overdose in first responders. However, fear of fentanyl exposure exists among both the public and professionals who handle drugs. Several studies have also suggested that fentanyl could potentially be used in chemical warfare or acts of terrorism.^3,5,6^ This fear can be leveraged by bad actors to incite mass panic among the public.

Currently, according to the CDC, in situations involving unknown powders, HazMat personnel are required to “determine the identity of the hazardous material before the *Unidentified Chemical* guideline is used”.^7^ While first responders take precautions in emergency situations such as wearing personal protective equipment (PPE) when testing unknown substances, rapid screening tools like fentanyl test strips (FTS) can inform responders of the next steps and the level of PPE required. FTS are lateral flow chromatographic immunoassay devices for qualitative detection of fentanyl.^8^ Although commercial FTS formulations are proprietary, they generally contain a sample pad, absorbent membrane, and test/control lines on a plastic backing.^9^ During FTS use, a liquid sample is applied to the sample pad and migrates along the absorbent membrane through capillary action. Target analytes then bind to antibodies (or equivalent biorecognition molecules) that produce a visible signal of binding.^10^ Control lines are included to ensure the FTS is working as expected. Results are interpreted through visual inspection for the presence or absence of colored lines.

FTS are used in a variety of applications, including urine drug testing, drug checking, and harm reduction. The CDC recommends the use of FTS as a harm reduction strategy aimed at reducing opioid overdose risk.^7,11–13^ A recent study found that legalizing FTS corresponds with a 7% decrease in overdose mortality, particularly by reducing unintentional drug overdose deaths.^14^ Emergency medical services (EMS) teams have also distributed FTS to the public for checking unknown powders in settings like music festivals and concert venues.^15^ The utility of FTS in harm reduction contexts suggests that they may also be valuable in emergency protocols. Since the strips help identify the potential contents of unknown substances, it is equally important to ensure they maintain accuracy in the presence of adulterants, diluents, and contaminants.

Much of the existing literature has focused on the efficacy of FTS strips with fentanyl analogs or derivatives,^16–18^ performance over time,^17^ or their ability to detect common adulterants.^16^ The strip being used has a limit of detection (LOD) of 0.20 μg/mL, but this does not account for interfering diluents. LOD is defined as the lowest concentration of a target drug that can be reliably detected.^19^ Diluents may be present at higher concentrations in cases involving chemical threats, where the intent is to create disruption or distress. While FTS are used in forensic settings as a certified secondary testing option,^16,20^ to our knowledge, no studies have applied test strips to chemical emergency response situations nor assessed how diluents may impact the established LOD. Given that these strips are increasingly used in harm reduction and distributed to the public, we must also consider their performance in such real-world scenarios.

## 2 Experimental Section

### 2.1 Materials

Fentanyl test strips were purchased from BTNX (Lot #DOAA2311775). A 1 mg/mL fentanyl certified reference material, obtained from Cayman Chemical (Ann Arbor, MI), was used to prepare stock solutions for the study procedures. HPLC-grade methanol, acquired from Thermo Fisher Scientific (Bridgewater, NJ), was used to dilute stock solutions to desired concentrations. Deionized (DI) water was prepared in-house by using an Elga PURELAB Ultra water purification system. Various household items, commonly found at grocery or drug stores, were selected to simulate diluents in “bad actor” scenarios. These included Baker’s Corner sugar, Welby ibuprofen (Lot #4DE1972A), Baker’s Corner All Purpose Flour, Kroger Corn Starch, Alleve (Lot #87380076), and King Arthur Gluten Free Measure for Measure Flour. Additional products included Extra Strength Tylenol (Lot #SAA026), Kirkland Signature Super B-Complex (containing B1, B2, niacin, B3, folate, B12, biotin, pantothenic acid, sodium, and potassium), Kirkland zinc supplements, Kroger Original Pancake and Waffle Mix, and Walgreens SmoothLax (polyethylene glycol; Lot #1104665).

### 2.2 Limit of Detection

To determine the LOD of the FTS the procedure began with the LOD on vendor’s packaging (0.20 μg/mL of fentanyl in DI water). Using a 1 mg/mL fentanyl certified reference material (HCl salt, with concentration reported as free base), a 10 μg/mL stock solution was prepared using HPLC-grade methanol. From this stock, test solutions were prepared at decreasing concentrations of fentanyl in DI water, starting at 0.20 μg/mL. That is, 100 μL of 10 μg/mL fentanyl in methanol stock solution was diluted to 5 mL with DI water to achieve a final concentration of 0.20 μg/mL fentanyl in DI water. The FTS were used in triplicate at each concentration. Results were interpreted as positive or negative based on the number of bands that appeared on the strip. One control band indicated a positive result for fentanyl, while two bands indicated a negative result, as shown in Figure 1. The FTS vendor also provides instructional videos on their Web site to help users perform the test accurately and consistently.^8^ If all three strips at a given concentration tested positive, the concentration was then decreased by increments of 0.05 μg/mL until at least two of three strips yielded a negative result. This process continued until 0.03 μg/mL, at which point all strips were negative. The last concentration where all three strips were clearly positive (0.05 μg/mL) was established as the experimental LOD. Notably, this value is four times lower than the reported LOD (0.20 μg/mL).^8^

**Figure 1:**
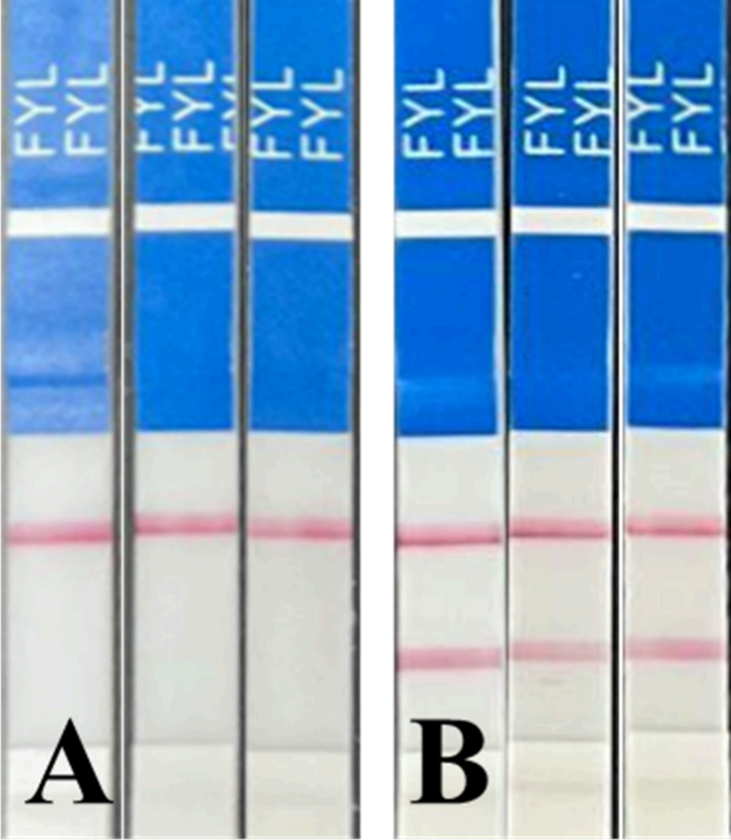
(A) Positive control (0.20 μg/mL fentanyl) and (B) negative control (DI water only) were tested to ensure that the intensity of the bands on the FTS was clearly visible for interpretation. For public and first responder use, each package of FTS includes an interpretation card to help users distinguish between positive, negative, and invalid results.

### 2.3 Test Strip Use

FTS vendor instructions were followed to maintain consistency and simulate real-life use scenarios. Although FTS use instructions are available online,^8^ specific measurement ranges and timing details are not precisely defined. Therefore, certain parameters (weight of powder and duration of strip immersion) were standardized in this study to ensure consistency. According to FTS vendor instructions, 5–10 mg of “unknown powder” is to be dissolved in 5 mL of DI water in a clear container and mixed until homogeneous. For this experiment, and to better reflect potential real-world applications, diluents were tested at slightly higher concentrations than those suggested by the vendor. This decision was based on the vague guidance provided (the vendor also states that a teaspoon of unknown powder is acceptable) and the realistic assumption that instruments such as analytical balances are not available in the field. Therefore, 15 mg of each diluent was used to better capture any potential false positives or interferences. This 15 mg aliquot of diluent was then dissolved into 5 mL of DI water (resulting in a 3 mg/mL concentration) with or without fentanyl.

Avoiding contact with the white section of the strip, the strip was dipped up to the blue line in the prepared solution for 10–15 s, then placed on a nonabsorbent surface for 5 min. Results were read after 5 min and not beyond 10 min, following the vendor’s guidelines. Test outcomes were interpreted according to the visual results shown in Figure 1, which are consistent with vendor instructions for determining the presence of fentanyl.

To better reflect real-world complexities, results were categorized into four outcome types: definite positive (Pos), definite negative (**Neg**), vague positive (*Pos’*), and vague negative (*Neg’*). While FTS use guides define a negative as the presence of two lines – regardless of line intensity – this approach, though helpful in harm reduction settings, may oversimplify interpretation in analytical contexts. Since the goal of this study was to determine the FTS LOD in the presence of various diluents, the faint appearance or disappearance of lines could indicate proximity to the detection threshold. Therefore, distinctions between definite and vague results were introduced to capture these nuances more accurately. These variations are shown in Figure 2A,B, where lines are more faintly visible than in Figure 1A,B demonstrating positive and negative controls. Therefore, this study defined a definite negative or positive as the clear presence of two or one lines, respectively. Vague positives were defined as a predominantly visible single line, while vague negatives indicate a possible, though faint, second line (see Figure 2). Solutions containing both diluents and fentanyl were tested until two of the three strips produced a definite positive result.

**Figure 2:**
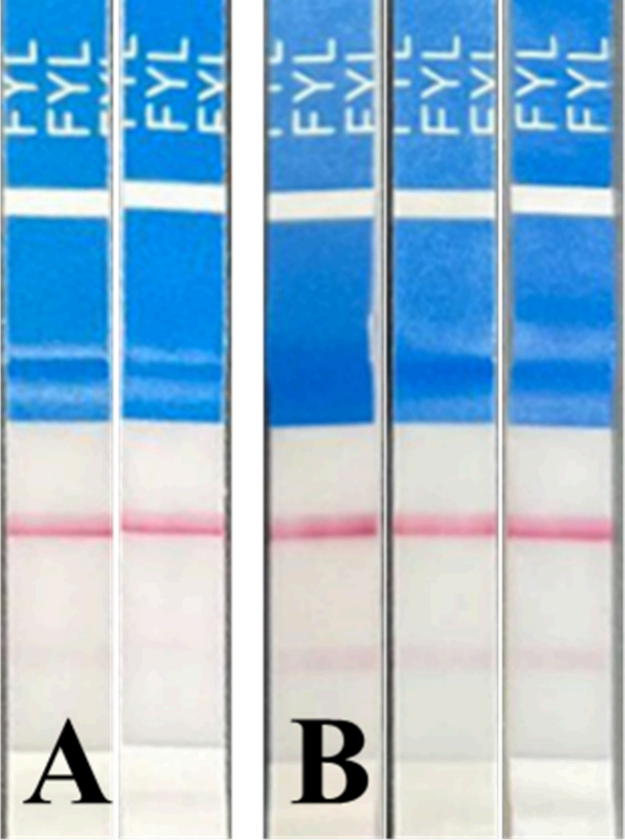
(A) FTS determined to be vaguely positive, as the second line appears faint upon visual inspection. (B) FTS labeled as vaguely negative, as the second line is barely visible even under optimal laboratory lighting conditions.

### 2.4 Diluent Interference

All diluents (15 mg) were tested independently (without fentanyl) in triplicate in 5 mL of water to determine if they produced false positives. Each FTS was dipped into the solution until fluid was visibly drawn onto the strip (indicated by a visual gradient). Although times varied slightly, strips were typically held in the solution for approximately 15 s. After dipping, each strip was placed on a clean, dry bench surface for 5 min. Strips were read and photographed at the 5 min mark for analysis. If strips were negative, a new solution was prepared containing both the diluent and fentanyl at the experimentally determined LOD.

Diluent (15 mg) was added to a 5 mL solution of DI water and fentanyl at the experimentally determined LOD of 0.05 μg/mL (or 0.25 μg fentanyl in 5 mL DI water). This produced a 60,000:1 ratio (by mass) of diluent to fentanyl, representing trace levels of fentanyl relative to the bulk mass of powder. This ratio was selected based on adoption of a standardized 15 mg aliquot of diluents and the experimentally derived FTS LOD in fentanyl-only solutions. The FTS was then dipped into the solution until a visual gradient was observed (10–15 s) and placed on the bench to dry for 5 min. After 5 min, results were read and documented. If all three strips tested positive, no further testing was conducted beyond the established LOD. However, if at least one strip was negative or vaguely negative, the fentanyl concentration was increased in increments of 0.05 μg/mL until all test strips produced a definite positive result. All strips and solutions were disposed of properly after testing.

## 3 Results and Discussion

The experimental LOD was determined to be 0.05 μg/mL of fentanyl, as described in Table 1. When testing the diluents alone in water, no solution produced a false positive result. However, it was determined that diluents in a fentanyl-water solution do affect the determined LOD of the FTS. Observed increases in FTS LOD are not a result of diluting the fentanyl concentration as solid diluent powders were added to 5 mL of prepared aqueous fentanyl solutions at described fentanyl concentrations. All diluents showed at least one vaguely negative result at 0.05 μg/mL (experimental LOD), and concentrations subsequently increased until an absolute positive result was attained (Table 2). Ibuprofen was the only diluent that required concentrations greater than 0.10 μg/mL of fentanyl to reach a positive result. While the precise mechanism behind the observed increases in LOD, particularly with ibuprofen, were not within the scope of this study, it is possible that insoluble diluent particles interfered with sample migration along the test strip or with antibody-analyte interactions at the test line. All diluents with fentanyl still performed better than the reported LOD of 0.20 μg/mL fentanyl.^8^

**Table 1:** Results as Positive (Pos) or Negative (**Neg**) from Determining the FTS Experimental LOD^a^

concentration of fentanyl (μg/mL)	strip 1	strip 2	strip 3
0.20	Pos	Pos	Pos
0.15	Pos	Pos	Pos
0.10	Pos	Pos	Pos
0.05	Pos	Pos	Pos
0.04	Pos	Pos	**Neg**
0.03	Pos	**Neg**	**Neg**

a Experiments conducted with decreasing concentrations of fentanyl (free base) in DI water, starting at the reported LOD (0.20 μg/mL). No diluents were present.

**Table 2:** Cumulative Results for Diluents and Fentanyl (Free Base) in DI Water^a^

diluent of interest (15 mg of each):	0.05 μg/mL fentanyl			0.10 μg/mL fentanyl			0.15 μg/mL fentanyl		
caffeine (15 mg)	Pos	*Neg’*	*Neg’*	Pos	Pos	Pos	NT	NT	NT
Corn Starch (15 mg)	*Neg’*	*Neg’*	*Neg’*	Pos	Pos	Pos	NT	NT	NT
Naproxen (15 mg)	*Neg’*	*Neg’*	*Neg’*	*Pos’*	Pos	Pos	NT	NT	NT
Gluten-free flour (15 mg)	*Neg’*	*Neg’*	*Neg’*	Pos	Pos	Pos	NT	NT	NT
Flour (15 mg)	*Neg’*	*Neg’*	*Pos’*	Pos	Pos	Pos	NT	NT	NT
Tylenol (15 mg)	*Neg’*	*Neg’*	*Neg’*	Pos	Pos	Pos	NT	NT	NT
Pancake Mix (15 mg)	*Neg’*	*Neg’*	*Neg’*	Pos	Pos	Pos	NT	NT	NT
Sugar (15 mg)	*Neg’*	*Neg’*	*Pos’*	Pos	Pos	Pos	NT	NT	NT
Zinc (15 mg)	*Neg’*	*Pos’*	Pos	Pos	Pos	Pos	NT	NT	NT
Vitamin B (15 mg)	*Neg’*	*Neg’*	*Neg’*	Pos	Pos	Pos	NT	NT	NT
Ibuprofen (15 mg)	*Neg*	*Neg*	*Pos’*	*Neg’*	*Pos’*	*Pos’*	Pos	Pos	Pos
Laxative (15 mg)	*Neg’*	*Neg’*	Pos	Pos	Pos	Pos	NT	NT	NT

a Diluent amounts remained constant (15 mg) and fentanyl concentration increased (0.05, 0.10, and 0.15 μg/mL) until FTS LOD was determined in diluent/fentanyl solutions. Addition of diluents reduced FTS sensitivity to fentanyl (increased LOD), most markedly in solutions of ibuprofen and fentanyl. Results reported as definite positive (Pos), vaguely positive (*Pos’*), vaguely negative (*Neg’*), definite negative (**Neg**), and not tested (NT, LOD determined halting further fentanyl concentration increases).

Interpretation of the test results revealed a significant challenge in this testing area. These test strips are designed to be accessible and user-friendly for groups that may not be in controlled lab environments or have immediate access to tools that could aid in result interpretation. Even under optimal laboratory conditions, positive/negative results were sometimes difficult to discern, particularly near the LOD. As seen in Figure 2 many results near the experimental LOD were interpreted as vague. For emergency personnel or the public who may rely solely on these strips for testing in time-sensitive situations, the issue of result interpretation remains a significant gap, especially with diluents involved. Nonetheless, it was observed that the FTS experimentally derived LOD of 0.05 μg/mL was lower than reported (0.20 μg/mL).

For future directions, this study provides a useful baseline for understanding how diluents interfere with FTS sensitivity. Mechanisms driving decreased FTS sensitivity in the presence of diluents could be identified through studies examining physical obstruction, nonspecific binding, or chemical interference with strip components. This knowledge can be applied to an expanded list of diluents, potentially including other drug test strips from other manufacturers. Additionally, these strips could play a role in harm reduction strategies used in chemical emergency response scenarios.

## 4 Conclusions

Several companies have developed immunoassay test strips to detect narcotic drugs are used in a variety of applications, ranging from harm reduction to clinical drug testing. These strips are believed to be a valuable secondary testing option in forensics cases involving unknown powders and may also be useful in chemical emergency response scenarios. Due to the rise of opioid addiction, overdose deaths, and resulting media coverage, fentanyl has gained notoriety and has been used (or its presence inferred) by bad actors to cause disruption and fear. However, unknown or unexpected powders may contain benign diluents such as sugar or flour. Given the frequent mixing of these substances, understanding whether diluents affect FTS sensitivity is crucial for their use in emergency scenarios.

This study is the first to apply test strips to chemical emergency response assessments of unknown substances and found that the presence of diluents does impact the LOD of the FTS. Although experimentally determined FTS LOD remained below the reported threshold of 0.20 μg/mL, even modest reductions in sensitivity may have operational implications for first responders triaging unknown powder incidents. In the early stages of a HazMat or chemical emergency response, FTS offer a practical screening tool: they are inexpensive, easy to use, provide rapid results, and do not require specialized training or instrumentation. These features make them well-suited for initial field-based assessments that inform downstream decisions about protective measures and resource deployment. However, because these strips are qualitative and susceptible to limitations such as reduced sensitivity in the presence of diluents and ambiguity in FTS result determination at the LOD, they should be used in conjunction with confirmatory testing protocols. This study highlights the importance of recognizing such limitations to ensure these tools are applied effectively and responsibly in real-world emergency contexts.

## Abbreviations

CCSA: Canadian Centre of Drug Abuse and Control
CDC: Center of Disease Control
DI: deionized
EMS: emergency medical services
FTS: fentanyl test strips
HazMat: hazardous materials response teams
HPLC: high-performance liquid chromatography
LOD: limit of detection
μg: microgram
mg: milligram
mL: milliliter
PPE: personal protective equipment
